# Disparities in Depressive Symptoms Between Heterosexual and Lesbian, Gay, and Bisexual Youth in a Dutch Cohort: The TRAILS Study

**DOI:** 10.1007/s10964-015-0403-0

**Published:** 2016-01-09

**Authors:** Chaïm la Roi, Tina Kretschmer, Jan Kornelis Dijkstra, René Veenstra, Albertine J. Oldehinkel

**Affiliations:** Department of Sociology, Interuniversity Center for Social Science Theory and Methodology (ICS), University of Groningen, Grote Rozenstraat 31, 9712 TG Groningen, The Netherlands; Department of Pedagogy and Educational Science, University of Groningen, Grote Rozenstraat 38, 9712 TJ Groningen, The Netherlands; Interdisciplinary Center Psychopathology and Emotion Regulation (ICPE), University Medical Center Groningen, Hanzeplein 1, 9713 GZ Groningen, The Netherlands

**Keywords:** Depressive symptoms, LGB youth, Minority stress, Pubertal development, Peer victimization, Parental rejection

## Abstract

Lesbian, gay, and bisexual (LGB) youth experience elevated levels of depressive symptoms compared to heterosexual youth. This study examined how differences in depressive symptoms between heterosexual and LGB youth developed from late childhood to early adulthood. The association between sexual orientation and depressive symptoms was estimated from age 11 to 22 using data from the TRacking Adolescents’ Individual Lives Survey, a longitudinal Dutch cohort study. Of the 1738 respondents (54.8 % girls) that provided information on sexual orientation, 151 self-identified as LGB. In line with the Minority Stress Framework, it was tested whether self-reported peer victimization and parental rejection mediated the association between sexual orientation and depressive symptoms. Results indicated that LB girls and bisexuals were at increased risk of depressive symptoms already at age 11. The difference increased over time and was related to pubertal development in girls and bisexual individuals. Furthermore, self-reported peer victimization (for both boys and girls), as well as parental rejection (for girls/bisexuals), mediated the association between sexual orientation and depressive symptoms. The authors conclude that already in late childhood, associations between sexual orientation and depressive symptoms are found, partly due to minority stress mechanisms.

**Electronic supplementary material:**

The online version of this article (doi:10.1007/s10964-015-0403-0) contains supplementary material, which is available to authorized users.

## Introduction

Sexual orientation has been linked to adolescent mental and physical health, with lesbian, gay and bisexual (LGB) adolescents faring worse than heterosexual adolescents (for recent reviews see Institute of Medicine 2011; Mustanski 2015). Depressive symptoms rank among the most frequently studied mental health outcomes related to sexual orientation (Almeida et al. 2009; Jiang et al. 2010; Ueno et al. 2014; Wang et al. 2014). Cross-sectional studies have found higher levels of depressive symptoms for LGB people in comparison to heterosexuals, in adolescence (Marshal et al. 2011) as well as adulthood (Institute of Medicine 2011; Meyer 2003). Longitudinal studies on the topic are scarce, with exceptions relying largely on data from the National Longitudinal Study of Adolescent to Adult Health (Add Health) (Fish and Pasley 2015; Marshal et al. 2013; Needham 2012). These studies found that, compared to heterosexual youth, same-sex or bisexually attracted youth experienced elevated levels of depressive symptoms in late adolescence (age 16), which persisted into early adulthood (age 29). What remains unclear, however, is (1) when disparities commence, (2) how they develop over time, and (3) what factors explain these disparities (Mustanski 2015). Aiming to fill these gaps, we examine from which developmental period disparities in depressive symptoms between heterosexual and LGB youth begin to occur and which factors act as catalysts of these disparities. Stigma and prejudice are arguably important antecedents of depressive symptoms in LGB people (Hatzenbuehler 2009; Meyer 2003). On the interpersonal level, LGB youth are at increased odds of being victimized by peers (Robinson et al. 2013; Williams et al. 2005) and of experiencing rejection by parents (Needham and Austin 2010; Pearson and Wilkinson 2013). Therefore, we study whether parental rejection and peer victimization mediate the potential association between sexual orientation and depressive symptoms.

The data used in the present study come from the TRacking Adolescents’ Individual Lives Survey (TRAILS), an ongoing prospective cohort study of Dutch youth that focuses on the development of mental health from childhood to adulthood (Oldehinkel et al. 2015). The Netherlands is generally thought of as an LGB-friendly country, known for its pro-gay legislation and relatively favorable public opinion about homosexuality (Lubbers et al. 2009; Takács and Szalma 2013; Van den Akker et al. 2013). One would thus expect that differences in health and well-being between heterosexual and LGB individuals are relatively small in the Netherlands. However, research on adults (Lewis 2009) as well as on adolescents (Kuyper 2015) found that Dutch LGB people experience disparities in health and well-being that are comparable to those found in other Western countries.

### Sexual Orientation and Depressive Symptoms in Adolescence

A substantial proportion of people suffers from depressive symptoms at some moment during adolescence (Saluja et al. 2004). Depressive symptoms thus inflict a serious burden on adolescent mental health. Moreover, depressive symptoms in adolescence can lead to impaired mental health in later life, as suffering from depressive symptoms in adolescence was found to increase the chance of developing a major depressive disorder in adulthood (Aalto-Setälä et al. 2002; Hill et al. 2014; Pine et al. 1999). Of particular interest to the current study is that depressive symptoms are more prevalent among LGB adolescents than among heterosexual adolescents (Kuyper 2015; Marshal et al. 2011; Mustanski 2015).

The Minority Stress Framework serves as an explanatory theoretical framework for such mental health disparities by sexual orientation (Meyer 2003) in stating that LGB people are regularly confronted with stigma and prejudice related to their sexual orientation. Both the stigma itself and fear of stigma can have a negative influence on LGB people’s health and well-being. Furthermore, stigma and prejudice are thought to obstruct the extent to which LGB individuals feel free to express themselves and their sexual orientation to others. Moreover, stigma and prejudice can elevate LGB people’s negative attitudes toward their own sexual orientation (internalized homophobia, Newcomb and Mustanski 2010). By contrast, ameliorating factors (e.g., an accepting family, gay-straight alliances in high school) might buffer the damaging effects that stigma and prejudice can have. From the minority stress framework we take the assertion that the social context is a heteronormative structure that can be prejudiced and stigmatizing toward LGB people (assumption 1). This stigmatization can increase the risk of depressive symptoms for LGB people in comparison to heterosexual people (assumption 2) (Hatzenbuehler 2009).

Susceptibility to LGB-related stigma presumably starts in the life phase during which LGB youth start to become aware of their sexual orientation. Studies on the development of (same-sex) sexual orientations suggested that the average age of self-awareness of one’s sexual orientation lies around 8–10 years (Maguen et al. 2002; Savin-Williams and Diamond 2000). According to Herdt and McClintock, sexual attraction starts to develop during adrenarche, which describes the development of the adrenal glands in middle to late childhood (Herdt and McClintock 2000; McClintock and Herdt 1996). Adrenarche is the biological process that underlies the start of the first phase of pubertal development. This first phase of puberty is characterized by a lack of external physical signs of puberty such as breast, genital or pubic hair development. It is only in later phases of puberty (driven by the start of other biological processes) that (the development of) primary and secondary sex characteristics become(s) visible (Dorn et al. 2006). If the start of sexual orientation development follows from adrenarche, the development of sexual orientation is thus already underway when children are in a developmental phase labelled prepubertal.

In line with the literature, we assume sexual orientation to follow a developmental process (Saewyc 2011). Pubertal development after adrenarche might stimulate this developmental process, as it has been found to serve as an important predictor for the onset of sexual activity and pre-coital sexual developments, such as sexual ideation and non-coital sexual behavior (Baams et al. 2015a; Halpern et al. 1993; Smith et al. 1985). Further pubertal development could therefore serve as an amplifier of the sexual orientation development that started with adrenarche, and so lead to an increase of the disparities in depressive symptoms between LGB youth and heterosexual youth, due to an intensification of susceptibility to stigma and prejudice toward LGB people.

We argue that susceptibility to LGB-related stigma and prejudice might follow from the awareness and development of one’s sexual orientation, by arguing that adrenarche and further pubertal development are indicators for the development of one’s sexual desires. However, sexual orientation is a multi-faceted concept that, apart from sexual desires, also encompasses romantic or affectional desires and self-identification (Diamond 2003; Savin-Williams 2006). Affectional desires might be driven by different biological processes than the ones that drive sexual desires (Diamond 2003). In addition, recognizing and acknowledging one’s sexual orientation might not only be influenced by biological processes, but also the societal context in which one is growing up. For instance, although beginning awareness of sexual orientation typically coincides with adrenarche, variation exists, with some people becoming aware of their sexual orientation before and some after late childhood (Maguen et al. 2002; Savin-Williams and Diamond 2000). Nonetheless, we envision adrenarche to function as a mechanism that might serve as a starting point for sexual orientation disparities between youth that identify as heterosexual and youth that identify as LGB.

We expect the development of an LGB sexual orientation to be linked to an increased risk of depressive symptoms, because LGB youth are confronted with stigma and prejudice related to their sexual orientation, resulting in minority stress (Meyer 2003). On the interpersonal level, peer victimization and parental rejection were often found to be important sources of minority stress (Birkett et al. 2015; Rothman et al. 2012). That is, studies have shown that sexual orientation victimization partially explains differences in depressive symptoms within samples of LGB youth (Baams et al. 2015b; Birkett et al. 2015). Furthermore, probability samples have repeatedly shown that LGB youth are at greater risk of being victimized by peers compared to heterosexual respondents, which partially explains sexual orientation differences in (mental) health (Bontempo and D’Augelli 2002; Robinson et al. 2013; Williams et al. 2005). Studies from the Netherlands have found evidence in favor of these mechanisms as well. Van Bergen et al. (2013) showed that high-school peer victimization was associated with higher rates of suicidal ideation and attempt within a sample of LGB adolescents. Furthermore, Dutch LGB youth experienced higher levels of victimization of homophobic name-calling and psychological distress (Collier et al. 2013; Van Beusekom et al. 2016).

Empirical evidence paints a similar picture with regard to parent–child relationships, another important source of stress within the minority stress framework. First, studies employing convenience samples from the US showed that parental rejection and parental support partly explained differences in psychological distress between LGB adolescents (Bouris et al. 2010; Puckett et al. 2015; Rothman et al. 2012; Ryan et al. 2009). Furthermore, studies on Add Health data suggested that (lack of) parental support partially mediates the association between same-sex attraction and decreased mental health (Needham and Austin 2010; Pearson and Wilkinson 2013; Teasdale and Bradley-Engen 2010). Within the Netherlands, similar mechanisms have been detected (Kuyper 2015; Van Bergen et al. 2013). In this study, we will also focus on the effect of peer victimization and parental rejection on depressive symptom levels of LGB youth and expect that these interpersonal mechanisms explain the association between sexual orientation and depressive symptoms at least partly.

### Differences Within the LGB Group

Thus far in our argument, we considered LGB adolescents to be a homogenous group, ignoring possible differences in sexual orientation disparities within the LGB group. Most prominently, differences could arise between boys and girls or between bisexuals and gays/lesbians. Although a meta-analysis on sexual orientation differences in depressive symptoms in adolescence found that gender did not moderate this association (Marshal et al. 2011), research has repeatedly shown that women experience elevated levels of depressive symptoms in comparison to men (e.g., Girgus and Yang 2015) and that girls develop an increased vulnerability for depressive symptoms compared to boys from early adolescence onwards (Oldehinkel et al. 2011; Petersen et al. 1991). This gender gap in depressive symptoms from early adolescence onwards has been related to a heightened affiliative need for girls in this developmental period (Cyranowski et al. 2000; Larson and Richards 1989). Personal characteristics that contrast group norms, such as a lesbian or bisexual orientation, might be particularly stressful for adolescent girls, as these may distort this heightened affiliative need. On the other hand, attitudes have been shown to be more negative toward GB men than toward LB women (Kite and Whitley 2003). Also, GB men are more frequently victimized and discriminated than LB women (Almeida et al. 2009; D’Augelli et al. 2002; Meyer et al. 2008), although this difference appears to be less pronounced in the Netherlands (Kuyper and Fokkema 2011). Thus, examining gender differences in the association between sexual orientation and depressive symptoms is worthwhile.

In addition, we examine whether the association between sexual orientation and depressive symptoms differs for bisexuals in comparison to gays/lesbians. There are several reasons why bisexual experiences may differ in salient ways from that of ‘monosexual’ (hetero- and homo-sexual) individuals, as bisexuals refuse dichotomous notions of gender and sexuality and acknowledge fluid desires (Carr 2006; Pramaggiore 2002). This could lead to bisexuality being perceived as something that does not exist, or an unstable combination of heterosexuality and homosexuality (Rust 2000, 2002). Empirical evidence with regard to differences between bisexual and gay/lesbian youth in terms of mental health problems is mixed. A meta-analysis by Marshal et al. (2011) led to the conclusion that bisexuality did not significantly moderate the association between sexual orientation and depressive symptoms in adolescence. Substantial variation between studies exists however, with some studies suggesting that bisexuals are at greater risk of mental health problems (Bostwick et al. 2010; Marshal et al. 2013) and some studies finding no statistically significant differences between bisexuals and gays/lesbians (Bostwick et al. 2014; Needham and Austin 2010). From both a theoretical and an empirical point of view, there are thus reasons to explore whether differences with heterosexuals in depressive symptoms are different for bisexuals than for gays and lesbians.

## Current Study

The aims of this study were to examine from what developmental period onwards disparities in depressive symptoms between heterosexual and LGB youth start to occur, how these disparities develop over time and what factors act as catalysts of these disparities. We argue that LGB youth begin to develop an increased risk of depressive symptoms from the period at which they start to become aware of their sexual orientation, as we expect them to experience a heightened susceptibility to LGB-related stigma and prejudice from that period onwards. We expect initial sexual orientation development to be stimulated at least partly by adrenarche, a bio-developmental process that occurs in late childhood. Therefore, our first hypothesis is that in late childhood, LGB youth already have higher levels of depressive symptoms than heterosexual youth (H1).

We furthermore assume sexual orientation to follow a developmental process (Saewyc 2011). Pubertal development after adrenarche might stimulate this developmental process, as it has been found to serve as an important predictor for the onset of sexual activity and pre-coital sexual developments, such as sexual ideation and non-coital sexual behavior (Baams et al. 2015a; Halpern et al. 1993; Smith et al. 1985). Further pubertal development could therefore serve as an amplifier of the sexual orientation development that started with adrenarche and increase the disparities in depressive symptoms between LGB youth and heterosexual youth through an intensification of susceptibility to stigma and prejudice toward LGB people. In short, we expect further pubertal development to lead to an increase in depressive symptom disparities between heterosexual and LGB youth (H2).

As argued above, we expect LGB youth to experience higher levels of depressive symptoms due to minority stressors and examined two highly salient types. Previous research in both the Netherlands as well as other countries found that LGB youth might fare worse than their heterosexual counterparts in terms of mental well-being, because they are more often subject to peer victimization (Baams et al. 2015b; Robinson et al. 2013; Van Beusekom et al. 2016). We will test this mechanism and expect that peer victimization mediates the association between sexual orientation and depressive symptoms (H3). Similarly, studies have found that LGB adolescents experience decreased mental well-being because they feel rejected by their parents more often than heterosexual adolescents (Kuyper 2015; Needham and Austin 2010). Based on this literature, we expect that parental rejection mediates the association between sexual orientation and depressive symptoms (H4).

This study adds to the literature by examining these mediating mechanisms by the time respondents are in late childhood. If we find evidence in favor of the presence of such mechanisms, this suggests that minority stress processes are already at work in that developmental period. To examine the developmental stability of associations, we additionally tested whether peer victimization in early adolescence (wave 2) and parental rejection in late adolescence (wave 4) mediated the association between sexual orientation and depressive symptoms. Lastly, this study will extensively explore potential gender differences and differences between bisexuals and gays/lesbians in the association between sexual orientation and depressive symptoms. Before estimating statistical models that serve to test our hypotheses formulated above, we therefore test whether boys and girls follow significantly different depressive symptom trajectories. Also, we check whether disparities in depressive symptom trajectories between LGB and heterosexual youth are different for boys and girls. Lastly, we explore whether contrasts to heterosexual youth in depressive symptoms are larger for bisexuals than for gays and/or lesbians. If substantial differences are found, we take this into account in further analyses.

## Data and Method

### Sample

We used data from the TRacking Adolescents’ Individual Lives Survey (TRAILS), an ongoing prospective cohort study of Dutch youth focused on the development of mental health from childhood to adulthood (Oldehinkel et al. 2015). Respondents were recruited between March 2001 and July 2002. *N* = 3145 children from 122 primary schools were approached for enrollment in the study. The sampling procedure consisted of two stages. First, five municipalities in the North of The Netherlands, including urban and rural areas, were requested to provide information from the community registers (i.e., name, date of birth, gender, address) of all inhabitants that were born between October 1 1989 and September 30 1990 (first two municipalities) or between October 1 1990 and September 30 1991 (last three municipalities). Subsequently, all primary schools in the five municipalities received a letter accompanied by detailed information about the goals, design and practical procedures of TRAILS. School participation was a prerequisite for eligible children and their parents to be approached. Secondly, parents/guardians were informed through information brochures about the study goals, selection procedure, confidentiality, and measures of the study, resulting in a baseline sample of *N* = 2230 respondents (response rate 76 %) (Huisman et al. 2008; de Winter et al. 2005). Extensive recruitment efforts have been made at baseline and throughout the study to prevent non-response bias (de Winter et al. 2005). Consequently, retention rates are fairly high (Oldehinkel et al. 2015), ensuring preservation of study outcomes. Five waves of data have currently been collected. We used data from all five waves (wave 1: *N* = 2230, *M* age = 11.1, 51 % girls; wave 2: *N* = 2149, *M* age = 13.6, 51 % girls; wave 3: *N* = 1816, *M* age = 16.3, 52 % girls; wave 4: *N* = 1881, *M* age = 19.1, 52 % girls; wave 5: *N* = 1778, *M* age = 22.3, 53 % girls).

### Measures

#### Dependent Variables

##### Depressive Symptoms

Depressive symptoms were assessed with the Youth Self Report (waves 1–3) and Adult Self Report (waves 4 and 5) (YSR/ASR), self-reported evaluations of emotional and behavioral problems in the past 6 months (Achenbach and Rescorla 2001). The 13 (YSR) or 14 (ASR) items of the Affective Problems scale reflect symptoms of a major depressive episode according to the DSM-IV (Achenbach and Rescorla 2003). Participants were asked to rate the items on a 3-point scale (0 = not true, 1 = a little or sometimes true, 2 = clearly or often true). The scale score reflects the mean score of the individual items. Twelve items appear on both the YSR and the ASR scale. The item “I sleep less than most boys and girls” appears in the YSR scale only. The items “I have the feeling that I can’t succeed” and “I find it difficult to take decisions” appear on the ASR scale only. Scale averages were created using the mean score on all items per wave. Note that models using scale scores based on only the twelve items that appeared in both the YSR and ASR provided very similar results to the ones we will present below (results available upon request). Cronbach’s α ranged between .72 (wave 2) and .84 (wave 4). Moreover, the instrument showed strong concurrent validity with DSM-IV Major Depressive Disorder (at wave 1) (van Lang et al. 2005).

#### Covariates

##### Sexual Orientation

Sexual orientation was measured using one item that assessed self-identified sexual orientation at wave 4 and wave 5. The question was phrased as follows: “What do you think you are? 1. Heterosexual 2. Gay/lesbian 3. Bisexual”. Respondents were coded as LGB if they self-identified as gay/lesbian or bisexual in one or both waves. Respondents that self-identified as gay/lesbian or bisexual in one of both waves, yet as heterosexual in the other, were coded as LGB. We not only fitted models where we collapsed the gay/lesbian category and bisexual category into one category labeled LGB, but also models where we differentiated between heterosexuals, lesbians/gays, and bisexuals. In these models, we recoded respondents from the LGB category as gay/lesbian when they self-identified as gay/lesbian in one or both waves.

As a robustness check, we re-estimated our models using two alternative operationalizations of sexual orientation. The alternative operationalizations pertained to respondents who self-identified as LGB in wave 4 and as heterosexual in wave 5. This answering pattern applied to 4 of the 58 boys (7 %) and 23 of the 93 girls (25 %) that were initially coded as LGB. In the first alternative operationalization, we coded the respondents with the aforementioned answering pattern as heterosexuals. In the second, we coded these respondents as missing. We re-estimated the models using the alternative operationalizations stratified by gender (results available upon request). Using these alternative operationalizations of sexual orientation did not lead to substantially different conclusions compared as the ones we will present, using the original operationalization.

##### Pubertal Development

Pubertal development was measured using the Pubertal Development Scale (PDS), a self-report measure of pubertal development. The scale was created as a non-invasive alternative for inferring pubertal development in research settings in which measures of pubertal development by means of physical examination are not feasible (Petersen et al. 1988). Research by Shirtcliff et al. (2009) showed that PDS scores were predictive of hormonal changes related to puberty in the same way as scores of a physical examination of pubertal status by trained nurse practitioners, ensuring validity of the PDS. The scale consisted of 5 sex-appropriate ordinal items measuring pubertal development on a 4-point scale, where scores of 1 refer to no pubertal development, whilst scores of 4 refer to completed development (Petersen et al. 1988). The PDS was measured at wave 2 and wave 3. The mean score of all PDS items per wave was used (Janssens et al. 2011).

##### Being Bullied

Being bullied was measured at wave 1, using a self-reported item on bullying. The item read as follows: “I am being bullied a lot”. Answering options were 0 “Not at all” 1 “A little or sometimes” and 2 “Clearly or often”. Answering options were dichotomized into 0 “Not bullied” and 1 “Bullied”, as additional analyses (available upon request) showed that the associations between self-identified bullying victimization and depressive symptoms were very similar for respondents that indicated to be bullied “A little or sometimes” and respondents that indicated to be bullied “Clearly or often”.

##### Relational Victimization

Relational victimization was measured using teacher reports of victimization to relational aggression by classmates at wave 2. Items included the following statement: “This student is the victim of gossip in the classroom.” Response options ran from 1 “(almost) never applicable” to 5 “(almost) always applicable”. A scale score was computed using the mean of three items. The scale showed very good reliability (α = .85).

##### Parental Rejection

Parental rejection was measured at waves 1 and 4, using self-reported parental rejection from the EMBU-C (Markus et al. 2003), a measure considered to be suitable for examining the perception of parenting styles in children (Markus et al. 2003) with confirmed factorial and construct validity (Deković et al. 2006). Respondents answered 4 questions on the extent to which they felt rejected by their father and/or mother, including items such as “Does your father/mother blame you for everything?” Response options ranged from 1 “No, never” to 4 “Yes, almost always”. We used the mean level of rejection experienced from both parents, if the respondents completed the measure for two parents. The mean scale score for one parent was used otherwise. The internal consistency of the scale was good at wave 1 (*α* = .84 for rejection by the father; *α* = 84 for rejection by the mother) and moderate at wave 4 (*α* = .70 for rejection by the father; *α* = 67 for rejection by the mother).

### Analysis

We estimated latent growth models to test our hypotheses (Muthén and Curran 1997), using Stata 13 (StataCorp LP 2013). In latent growth models, latent intercept and slope factors are created that serve to explain the overall pattern in the data. They consist of both a fixed mean effect and a random effect, which represents the amount of variance around this mean effect (Acock 2013). Models were estimated using Full Information Maximum Likelihood in order to compensate for missing data (Allison 2003; Enders and Bandalos 2001). As the Affective Problems scale was relatively skewed and the residuals of the estimates in a baseline model seemed to be somewhat skewed and leptokurtic (details available upon request), we used robust standard errors when estimating the models.

**Fig. 1:**
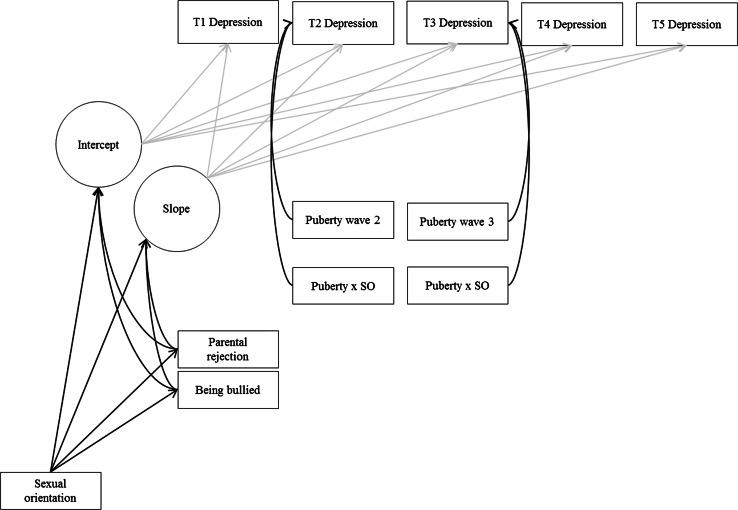
Statistical model

The first hypothesis was tested by estimating whether an LGB sexual orientation had a significantly positive effect on the mean intercept. Hypothesis 2 was tested by adding an interaction effect between an LGB sexual orientation and pubertal development at wave 2 and 3 on depressive symptoms at wave 2 and 3. A positive interaction effect suggests an increase of depressive symptom disparities. Time-varying covariates serve to explain variance in depression scores that are not already explained by the overall trajectories, which are captured by the latent intercept and slope factors (Acock 2013). Hypotheses 3 and 4 were tested by estimating indirect effects of an LGB sexual orientation on the intercept and slope of the depressive symptom trajectories via peer victimization (Hypothesis 3) and parental rejection (Hypothesis 4). A product of coefficients method was chosen to assess the significance of the indirect effects (Preacher and Hayes 2008). As recommended in the literature, we allowed residual variances of the mediators (peer victimization and parental rejection) to co-vary (Preacher and Hayes 2008). A graphical representation of our statistical model is shown in Fig. 1. In addition to the model portrayed in Fig. 1, we estimated models where we also included relational victimization at wave 2 and parental rejection at wave 4 and estimated whether these variables mediated either the association between sexual orientation and depressive symptoms at wave 3 (for wave 2 relational victimization) or wave 5 (for wave 4 parental rejection). Because we found no evidence pointing to such mechanisms, the results of these models will be reported only briefly (detailed results available upon request).

As stated above, we anticipated the association between sexual orientation and depressive symptoms to differ between boys and girls and between gays/lesbians and bisexuals. Therefore, after ascertaining that depressive symptom trajectories differed between boys and girls, we estimated models stratified by gender, as well as a model where gays/lesbians and bisexuals were examined as separate groups. For each subgroup, we fitted two models. In the first model, depressive symptom disparities were estimated using a latent intercept and latent linear slope factor. Sexual orientation was added to this model as a time-constant covariate to explain differences in the intercept and slope. This model tested hypothesis 1. Subsequently, a second model was estimated where we added the effect of peer victimization and parental rejection on the intercept and slope, the effect of sexual orientation on peer victimization and parental rejection, as well as the effect of pubertal development and the interaction between pubertal development and an LGB sexual orientation on depressive symptoms at wave 2 and 3. We only included a linear slope, because models with a quadratic slope returned non-significant quadratic effects (for the models on girls and the effect of bisexuality), or did not converge (for the model on boys).

### Propensity Score Matching

The group of LGB respondents in our sample was relatively small. Therefore it is possible that differences between LGB and heterosexual respondents resulted from chance concentrations of background factors that enhance the probability of depressive symptoms, yet are unrelated to one’s sexual orientation and the stigma and prejudice related to it. In order to eliminate this possibility, we employed propensity score matching. This is a method that aims to balance the distribution of covariates in the group of LGB youth (“treated”) and the group of heterosexual youth (“control”) (Stuart 2010). LGB respondents were matched to heterosexual respondents with similar scores on a group of background characteristics measured at the first wave, or retrospective accounts of characteristics of the respondent’s life that predated wave one. Background matching variables included parental socio-economic status, perinatal complications, negative childhood events (e.g. death of a household member, severe illness of sibling), long-term difficulties (e.g. chronicle disease of respondent or household member, protracted conflicts between family members), early childhood (age 0–5) stressfulness of life, intelligence, and depressive symptom levels of the respondents’ parents. For a detailed description of the matching variables, the exact matching procedure and the achieved balance after matching, please see appendix A.

Propensity score estimates were used to estimate the probability of being LGB on the basis of scores on the matching variables, for all 1738 respondents for whom information on sexual orientation was available. Multiple neighbors within caliper matching with resampling of matched control (heterosexual) cases was used. Because there were more than 11 heterosexual respondents for every LGB respondent, we allowed for up to 10 potential neighbors for every LGB respondent. That is, LGB respondents were matched with up to 10 heterosexual respondents, as long as there were 10 heterosexual respondents that were similar to them in terms of scores on the matching variables. When choosing a caliper, we sought for a caliper size that allowed to achieve balance without losing substantial numbers of LGB respondents due to absence of heterosexual respondents that were similar enough to them (Morgan and Harding 2006). A caliper of 0.025 points difference on the propensity score fulfilled this aim. Our analyses were consequently performed on the matched and weighted groups (Wu et al. 2008, 2010).

Differences in standardized propensity scores between LGB and heterosexual respondents were moderate, yet highly statistically significant before matching (boys: −0.36, *p* < .001; girls: −0.65, *p* < .001; bisexuals vs. heterosexuals: −0.66, *p* < .001). After matching, differences in standardized propensity scores were close to zero and non-significant (boys: −0.02, n.s.; girls: −0.01, n.s.; bisexuals vs. heterosexuals: −0.01, n.s.). This suggests that balance between our LGB respondents and the matched heterosexual respondents was achieved, and that differences with regard to depressive symptoms and explanatory mechanisms for these disparities cannot be attributed to differences in the matching variables (Stuart 2010). After the matching procedure, 57 GB boys were matched with 380 heterosexual boys, 90 LB girls were matched with 486 heterosexual girls, and 112 bisexual adolescents were matched with 744 heterosexual adolescents.

Standardized propensity scores were included as time-constant covariates on the intercept and slope in models of depressive symptom trajectories to further adjust for small differences that could remain after matching (Ho et al. 2007). The matching procedure prevented us from assessing model fit using traditional model fit measures such as the Root Mean Square Error of Approximation (RMSEA) and the Comparative Fit Index (CFI) (Bentler 1990; Browne and Cudeck 1993), as weighting was employed in order to achieve balance between the propensity scored LGB and matching heterosexual respondents. Consequently, model coefficients were estimated using robust standard errors and a pseudo-log-likelihood substituted the log-likelihood function for achieving model convergence (StataCorp LP 2013).

## Results

### Descriptive Statistics

Table 1 shows the frequencies of the sexual orientation variable for boys and girls. A total of 8.7 % of our respondents self-identified as LGB, which is roughly similar to other population estimates of the proportion of LGB people (Herbenick and Reece 2010; Kuyper 2006; Mosher et al. 2005). Furthermore, girls mostly self-identified as bisexual when they did not self-identify as heterosexual, whereas such an association did not seem to be present for boys. Such a pattern in responses is not uncommon in studies that measure self-identified sexual orientation in late adolescence or early adulthood (Bostwick et al. 2010; Marshal et al. 2013). Table 2 presents descriptive statistics by wave. The average depressive symptoms score over all observations was 0.29 (*SD* = 0.28). Depressive symptoms scores seem rather stable on average. Almost one-third of the respondents self-identified as a victim to bullying at wave 1.

**Table 1:** Self-identified sexual orientation by gender

	Heterosexual	Gay/Lesbian	Bisexual	Total
Boys	727 (92.61 %)	27 (3.44 %)	31 (3.95 %)	785
Girls	860 (90.24 %)	12 (1.26 %)	81 (8.50 %)	953
Total	1587 (91.31 %)	39 (2.24 %)	112 (6.44 %)	1738

Observed counts and row percentages

Row percentages might not sum to 100 due to rounding

**Table 2:** Descriptive statistics by wave for the whole sample

Variable (range)	Wave				
	1	2	3	4	5
Depressive symptoms (0–1.86)	0.29 (0.25)	0.27 (0.26)	0.30 (0.27)	0.30 (0.30)	0.31 (0.31)
Pubertal development (0–3)	–	1.41 (0.67)	2.24 (0.51)	–	–
Self-reported bullying victimization (0–1)	0.32 (701)	–	–	–	–
Relational victimization reported by teacher (1–5)	–	1.39 (0.60)	–	–	–
Parental rejection	1.48 (0.31)	–	–	1.46 (0.41)	–

Observed count added to proportion being bullied within parentheses

### Differences Within the LGB Group

**Fig. 2:**
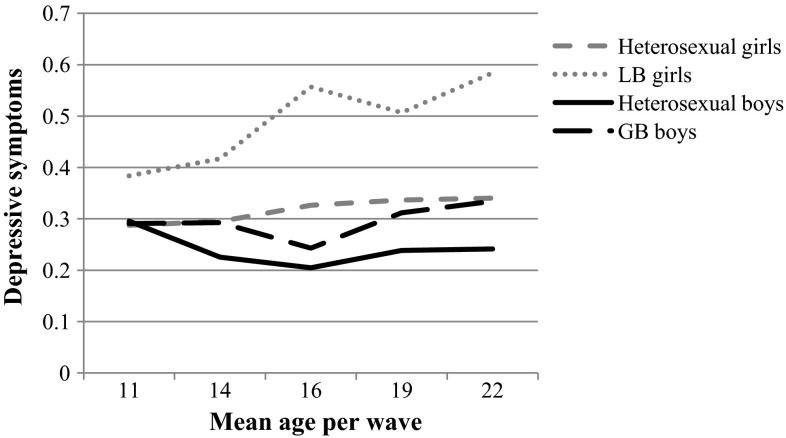
Depressive symptoms by sexual orientation and gender

As stated above, we examined differences in associations between sexual orientation and depressive symptoms between boys and girls, as well as between bisexuals and gays/lesbians. As an empirical justification for this objective, we estimated a preliminary latent growth model where we compared the mean intercept and slope for boys and girls. Furthermore, we provide descriptive information on depressive symptom trajectories by sex and sexual orientation in Fig. 2. Figure 2 indicates that LGB youth had a higher risk of depressive symptoms in comparison to heterosexuals. Additionally, discrepancies between LGB and heterosexual youth appear larger for girls than for boys. Moreover, Fig. 2 suggests that the development of depressive symptoms follows a different pattern for boys and for girls. A group comparison indicated that boys and girls indeed had a significantly different intercept (*χ2* (1) = 22.53, *p* < .001.) and slope (*χ2* (1) = 64.60, *p* < .001).

**Fig. 3:**
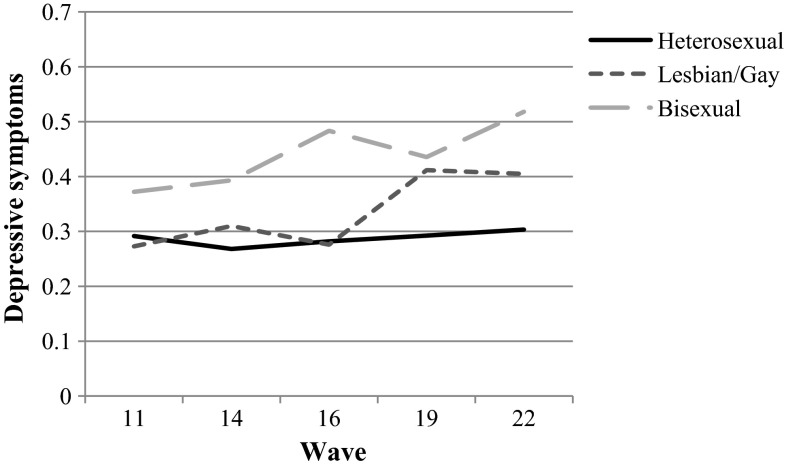
Depressive symptoms for heterosexuals, gays/lesbians and bisexuals

Figure 3 shows that discrepancies in depressive symptoms were larger for bisexuals compared to heterosexuals, than for gays and lesbians compared to heterosexuals, especially in waves one to three. The larger discrepancies for bisexuals might reflect that most respondents who self-identified as bisexual were girls. In sum, these preliminary analyses provided an empirical justification for our intention to examine differences in the association between sexual orientation and depressive symptoms between boys and girls, as well as between gays/lesbians and bisexuals. In the following, we present the results of models stratified by gender, as well as a model where gays/lesbians and bisexuals were examined as two separate groups.

### Latent Growth Models

#### Boys

Results for our latent growth models for boys are displayed in Table 3. Model one indicates that GB boys did not have significantly higher intercept levels in depressive symptoms than heterosexual boys [*b* = 0.02(0.04), n.s.], lending no support to hypothesis 1. Furthermore, no significant slope differences between GB and heterosexual boys were found [*b* = 0.02(0.01), n.s.]. We thus did not find that GB boys displayed higher levels of depressive symptoms than heterosexual boys in late childhood, or that they developed higher levels of depressive symptoms compared to heterosexual boys over time.

**Table 3:** Latent growth model depressive symptom disparities (boys only)

Direct effects	Model 1	Model 2
	B (SE)	B (SE)
*Intercept*		
Sexual orientation	0.02 (0.04)	−0.02 (0.03)
Standardized propensity score	0.03 (0.02)	0.01 (0.02)
Being bullied		0.19 (0.04)***
Parental rejection		0.15 (0.06)***
Constant	0.25 (0.02)***	0.21 (0.02)***
*Slope*		
Sexual orientation	0.02 (0.01)	0.03 (0.01)*
Standardized propensity score	−0.01 (0.01)	−0.01 (0.01)
Being bullied		−0.05 (0.01)***
Parental rejection		0.002 (0.021)
Constant	−0.005 (0.005)	0.01 (0.01)
*Being bullied*		
Sexual orientation		0.19 (0.07)**
*Parental rejection*		
Sexual orientation		−0.02 (0.05)
*Depressive symptoms wave 2*		
Pubertal development		−0.04 (0.01)**
Pubertal development × LGB		0.02 (0.02)
*Depressive symptoms wave 3*		
Pubertal development		−0.02 (0.01)***
Pubertal development × LGB		−0.002 (0.018)
*Indirect effects*		
Sexual orientation → being bullied → intercept		0.04 (0.01)*
Sexual orientation → being bullied → slope		−0.009 (0.004)*
Sexual orientation → parental rejection → intercept		−0.003 (0.007)
Sexual orientation → parental rejection → slope		−0.00004 (0.0005)

*N* = 437; 57 GB boys and 380 heterosexual boys

Unstandardized effects. Robust standard errors in parentheses

^ǂ^ *p* < .10; * *p* < .05; ** *p* < .01; *** *p* < .001

In model two, we did not find that pubertal development was associated with increased depression disparities between GB and heterosexual boys [wave 2: *b* = 0.02(0.02), n.s.; wave 3: *b* = 0.002(0.018), n.s.], lending no support to hypothesis 2. We did however find sexual orientation to be indirectly related to higher intercept levels of depressive symptoms via bullying victimization [*b* = 0.04(0.01), *p* < .05]. GB boys reported a higher prevalence of bullying victimization [*b* = 0.19(0.07), *p* < .01], whilst bullying victimization was related to higher intercept levels of depressive symptoms [*b* = 0.19(0.04), *p* < .001]. These results were in line with hypothesis 3. Furthermore, sexual orientation had an indirect negative effect on the slope of depressive symptoms [*b* = −0.009(0.004), *p* < .05]. This means that the indirect intercept differences in depressive symptoms due to wave one bullying victimization were attenuated over time. In contrast to model one, a direct association between a GB sexual orientation and the slope of depressive symptom levels was found [*b* = 0.03(0.01), *p* < .001] in model two, suggesting that GB boys experienced increased levels of depressive symptoms over time, compared to heterosexual boys. No evidence in favor of an indirect association between a GB sexual orientation and depressive symptoms via parental rejection was found [intercept: *b* = −0.003(0.007), n.s.; slope: *b* = −0.00004(0.0005), n.s.], lending no support to hypothesis 4.

In addition to the model portrayed in Table 3, we estimated models where we also included relational victimization at wave 2 and parental rejection at wave 4 and estimated whether these variables mediated either the association between sexual orientation and depressive symptoms at wave 3 (for wave 2 relational victimization) or wave 5 (for wave 4 parental rejection). None of these indirect effects reached statistical significance (detailed results available upon requests).

#### Girls

Results for our latent growth models on girls are displayed in Table 4. Model one indicates that LB girls had significantly higher intercept levels of depressive symptoms than heterosexual girls [*b* = 0.10(0.04), *p* < .01], consistent with hypothesis 1. Furthermore, significant slope differences between LB and heterosexual girls were found [*b* = 0.03(0.01), *p* < .05]. This means that LB girls experienced increased levels of depressive symptoms over time compared to heterosexual girls, in addition to the observed intercept differences in late childhood.

**Table 4:** Latent growth model depressive symptom disparities (girls only)

Direct effects	Model 1	Model 2
	B (SE)	B (SE)
*Intercept*		
Sexual orientation	0.10 (0.03)**	0.05 (0.03)^ǂ^
Standardized propensity score	0.03 (0.01)*	0.03 (0.01)*
Being bullied		0.13 (0.03)***
Parental rejection		0.28 (0.07)***
Constant	0.27 (0.01)	0.25 (0.02)***
*Slope*		
Sexual orientation	0.03 (0.01)*	0.04 (0.01)**
Standardized propensity score	−0.009 (0.005)*	−0.009 (0.004)*
Being bullied		−0.03 (0.01)*
Parental rejection		−0.02 (0.02)
Constant	0.021 (0.005)***	0.03 (0.01)***
*Being bullied*		
Sexual orientation		0.15 (0.06)*
*Parental rejection*		
Sexual orientation		0.09 (0.04)*
*Depressive symptoms wave 2*		
Pubertal development		0.002 (0.006)
Pubertal development × LGB		−0.01 (0.02)
*Depressive symptoms wave 3*		
Pubertal development		0.006 (0.005)
Pubertal development × LGB		0.03 (0.02)^ǂ^
*Indirect effects*		
Sexual orientation → being bullied → intercept		0.02 (0.01)*
Sexual orientation → being bullied → slope		−0.004 (0.003)
Sexual orientation → parental rejection → intercept		0.02 (0.01)*
Sexual orientation → parental rejection → slope		−0.002 (0.002)

*N* = 576; 90 LB girls and 486 heterosexual girls

Unstandardized effects. Robust standard errors in parentheses

^ǂ^ *p* < .10, * *p* < .05; ** *p* < .01; *** *p* < .001

In model two, we found that pubertal development was marginally associated with depression disparities between LB and heterosexual girls at wave 3 [*b* = 0.03(0.02), *p* = .057], in line with hypothesis 2. Pubertal development thus increased the differences in depressive symptoms between LB and heterosexual girls that were already present in late childhood. We furthermore found sexual orientation to be indirectly related to higher intercept levels of depressive symptoms via bullying victimization [*b* = 0.02(0.01), *p* < .05]. These results were in line with hypothesis 3. In addition, results pointed to an indirect association between an LB sexual orientation and higher intercept levels of depressive symptoms via parental rejection [*b* = 0.02(0.01), *p* < .05], consistent with hypothesis 4. In comparison to model one, the direct association between an LB sexual orientation and depressive symptom intercept levels decreased from .10 to .05 (*p* = .074) in model two.

In addition to the model portrayed in Table 4, we estimated models where we also included relational victimization at wave 2 and parental rejection at wave 4 and estimated whether these variables mediated either the association between sexual orientation and depressive symptoms at wave 3 (for wave 2 relational victimization) or wave 5 (for wave 4 parental rejection). No evidence was found pointing to such mechanisms (detailed results available upon requests).

#### Heterosexuals Versus Bisexuals

The small size of the group of participants within the LGB group that self-identified as gay/lesbian (*n* = 39) is likely to lead to problems with regard to power, model convergence, and bias in parameter estimates (Muthén and Curran 1997). Moreover, as our descriptive analyses showed that the differences in terms of depressive symptoms were larger for the bisexual group than the gay/lesbian group, we fitted a model where we compared the heterosexual group with the bisexual group and excluded the gay/lesbian group from these analyses.

Results for our latent growth models on the association between bisexuality and depressive symptoms are displayed in Table 5. Model one indicates that bisexuals had significantly higher intercept levels in depressive symptoms than heterosexuals [*b* = 0.09(0.03), *p* < .01], in line with hypothesis 1. Furthermore, significant slope differences between bisexuals and heterosexuals were found [*b* = 0.03(0.01), *p* < .05] suggesting that bisexuals experienced increased levels of depressive symptoms over time compared to heterosexuals, in addition to the observed intercept differences.

**Table 5:** Latent growth model depressive symptom disparities (bisexuals)

Direct effects	Model 1	Model 2
	B (SE)	B (SE)
*Intercept*		
Sexual orientation	0.09 (0.03)**	0.04 (0.03)
Standardized propensity score	0.04 (0.01)***	0.04 (0.01)***
Being bullied		0.14 (0.03)***
Parental rejection		0.23 (0.05)***
Constant	0.26 (0.01)***	0.23 (0.01)***
*Slope*		
Sexual orientation	0.03 (0.01)*	0.03 (0.01)**
Standardized propensity score	0.002 (0.004)	0.002 (0.004)
Being bullied		−0.04 (0.01)**
Parental rejection		−0.02 (0.02)
Constant	0.004 (0.004)	0.014 (0.005)**
*Being bullied*		
Sexual orientation		0.17 (0.05)**
*Parental rejection*		
Sexual orientation		0.08 (0.04)*
*Depressive symptoms wave 2*		
Pubertal development		0.004 (0.007)
Pubertal development × LGB		−0.004 (0.017)
*Depressive symptoms wave 3*		
Pubertal development		0.004 (0.004)
Pubertal development × LGB		0.02 (0.01)^ǂ^
*Indirect effects*		
Sexual orientation → being bullied → intercept		0.02 (0.01)**
Sexual orientation → being bullied → slope		−0.006 (0.003)*
Sexual orientation → parental rejection → intercept		0.02 (0.01)*
Sexual orientation → parental rejection → slope		−0.002 (0.001)

*N* = 856; 112 bisexual youth and 744 heterosexual youth

Unstandardized effects. Robust standard errors in parentheses

^ǂ^ *p* < .10; * *p* < .05; ** *p* < .01; *** *p* < .001

In model two, we found that pubertal development was marginally associated with depression disparities between bisexuals and heterosexuals at wave 3 [*b* = 0.02(0.01), *p* = .084], in line with hypothesis 2. Pubertal development thus increased the differences in depressive symptoms between bisexuals and heterosexuals in late childhood. We furthermore found bisexuality to be indirectly related to higher intercept levels of depressive symptoms via bullying victimization [*b* = 0.02(0.01), *p* < .01]. Bisexuals reported a higher prevalence of bullying victimization [*b* = 0.17(0.05), *p* < .01], whilst bullying victimization was related to higher intercept levels of depressive symptoms [*b* = 0.14(0.03), *p* < .001]. These results were in line with hypothesis 3. Furthermore, bisexuality had a significant indirect negative effect on the slope of depressive symptoms [*b* = −0.006(0.003), *p* < .05]. That is, the indirect intercept differences in depressive symptoms due to wave one bullying victimization were attenuated over time. In addition, results pointed to an indirect association between bisexuality and higher intercept levels of depressive symptoms via parental rejection [*b* = 0.02(0.01), *p* < .05], consistent with hypothesis 4. In comparison to model one, the direct association between an LGB sexual orientation and depressive symptom intercept levels decreased from .09 to .04 in model two, and was no longer significant.

Lastly, we estimated models where we also included relational victimization at wave 2 and parental rejection at wave 4 and estimated whether these variables mediated either the association between bisexuality and depressive symptoms at wave 3 (for wave 2 relational victimization) or wave 5 (for wave 4 parental rejection). None of these indirect effects reached statistical significance (detailed results available upon requests).

## Discussion

LGB youth experience elevated levels of depressive symptoms compared to heterosexual youth (Marshal et al. 2011; Wang et al. 2014). The Minority Stress Framework (Meyer 2003) serves as an explanatory framework for such disparities and states that they are the results of stigma and prejudice related to an LGB sexual orientation. Yet, information on the development of depressive symptom disparities over time is scarce (Mustanski 2015). We tried to fill this gap by estimating depressive symptom disparities between heterosexual and LGB youth in a Dutch cohort sample from age 11 to 22. We did so by establishing whether the LGB youth in our sample experienced elevated levels of depressive symptoms compared to heterosexual youth already at age 11, and whether we could find evidence in favor of the minority stress framework at that age. To address this aim, we focused on two potential sources of minority stress at the interpersonal level, peer victimization and parental rejection (Pearson and Wilkinson 2013; Robinson et al. 2013). Special attention was payed to potential gender differences in the effect of sexual orientation, as well as potential differences between bisexual and gay/lesbian youth in depressive symptom disparities.

Preliminary analyses indicated that men and women followed different depression trajectories. Furthermore, preliminary analyses suggested that sexual orientation disparities in depressive symptoms were substantially larger for girls than for boys. We therefore stratified our analyses by gender. In these stratified analyses we found that already at age 11, LB girls were at an increased risk of depressive symptoms compared to heterosexual girls. Results furthermore indicated that these differences increased over time and were related to pubertal development. The intercept differences in depressive symptoms by sexual orientation were partially mediated by self-identified peer victimization, as well as parental rejection. For girls, we were thus able to detect mechanisms in line with the Minority Stress Framework, already at age 11. Contrary to LB girls, no intercept differences in depressive symptoms were found for GB boys compared to heterosexual boys. For boys, we did however detect an indirect effect of sexual orientation on depressive symptoms, via self-reported peer victimization. Moreover, descriptive analyses suggested that sexual orientation disparities were larger for bisexuals than for gays/lesbians. We therefore fitted an additional latent growth model, where we focused on the differences in depressive symptoms between heterosexuals and bisexuals. In this model we found that already at age 11, bisexuals experienced an elevated risk of depressive symptoms compared to heterosexuals. Results further indicated that these differences increased over time and were related to pubertal development. The intercept differences in depressive symptoms by sexual orientation were partially mediated by self-identified peer victimization, as well as parental rejection. Also for bisexuals, we were thus able to detect mechanisms in line with the Minority Stress Framework, already at age 11.

Previous research on adolescents did not find that differences in depressive symptoms between LGB and heterosexual youth were larger for girls than for boys (Marshal et al. 2011). Yet, disparities in our sample were more pronounced for girls than for boys. One explanation could be that during adolescence, when girls start to develop extra vulnerability for depressive symptoms, not conforming to the group norm of heterosexuality is particularly aggravating, as it may distort the heightened affiliative need that girls develop in adolescence (Cyranowski et al. 2000), and so further enhance their already increased vulnerability for depressive symptoms. This heightened affiliative need in girls in comparison to boys might also explain why we found an indirect association between sexual orientation and depressive symptoms via parental rejection for girls only. That is, both GB boys and LB girls displayed higher levels of parental rejection in comparison to their heterosexual counterparts, yet only in LB girls this also led to higher levels of depressive symptoms.

Similarly, previous research in adolescents did not find that bisexual youth showed larger differences in depressive symptoms compared to heterosexual youth, than gay or lesbian youth (Marshal et al. 2011). Bisexual youth did however seem to experience larger depression disparities than heterosexual youth, in comparison to gay/lesbian youth. A lack of collective self-esteem in bisexual youth could account for this finding. The social status of bisexuals has been described as one of “double marginality”, meaning that they feel a lack of identification with both heterosexuals and homosexuals (Weinberg et al. 1994). This is reflected in studies that discussed bisexual women’s distinctive experiences with discrimination. For instance, research in adult populations has found bisexual women to report higher levels of discrimination than lesbians in queer settings (but lower levels in straight ones) (Carr 2011; Kuyper and Fokkema 2011). Similarly, studies have found that bisexuals experience significantly less social identification with LGB people and were less inclined to participate in LGB activism than lesbians and gays (Cox et al. 2010; Friedman and Leaper 2010).

This study is not without limitations. A lot of our reasoning is based on the assumption that the increased risk of depressive symptoms for LGB youth was a result of prejudiced and stigmatizing experiences of these youth related to their sexual orientation. One could argue that in order for such experiences to occur, LGB individuals should have an outwardly recognizable lesbian, gay, or bisexual orientation. For instance, we observed higher rates of self-reported peer victimization and parental rejection amongst our respondents yet cannot be sure that these differences have anything to do with sexual orientation. That is, we do not know whether or not the respondents that self-identified as LGB in our study were “out”. The importance of being out for LGB victimization to occur, however, can be questioned. A recent study on an LGB sample found that others’ perceived knowledge of the respondents’ sexual identity was only weakly associated with depressive symptoms and sexual orientation victimization (Baams et al. 2015b), suggesting that being out is hardly associated with depressive symptom levels. Also, a recent study showed that attempts of LGB adolescents to hide their sexual orientation in order to avoid sexual orientation victimization were unsuccessful (Russell et al. 2014). Lastly, it has been found that coming out by LGB youth can have adverse effects, such as negative reactions by the family or increased risks of peer victimization (Institute of Medicine 2011). A second limitation relates to our finding that the association between sexual orientation and depressive symptoms seemed to be more pronounced for bisexuals/LB girls. We were not able to test whether this was due to the fact that the association was larger for LB girls than for GB boys, or whether the association was larger for bisexuals than for gays and lesbians. The group of lesbian girls in our sample was too small to generate reliable estimates for such a test (*n* = 12). Related to this, the operationalization of sexual orientation in our sample was suboptimal, because the three answering options represent a fairly limited notion of the concept of sexual orientation, and the item only reflects the self-identification dimension of the multidimensional construct that sexual orientation is (Savin-Williams 2006). Lastly, because of the large amount of statistical tests conducted in this study, some of our findings may be a consequence of Type I error(s). Relatedly, the size of our sample provided us with limited power in light of the complex statistical models employed. This could have caused us to miss relevant associations due to Type II error(s).

Further research on the topic is needed. First of all, although this study had the opportunity to study the topic of well-being of LGB youth using a unique longitudinal dataset, the number of respondents that self-identified as lesbian, gay, or bisexual was not very high. This might have affected the robustness of our findings. Further research is thus needed to examine whether the mechanisms that we found to be present at late childhood, can be corroborated using other data. Additionally, we found that self-reported levels of peer victimization mediated the association between sexual orientation and depressive symptoms. Teacher-reports of relational victimization did however not mediate this association (although our LGB-respondents reported higher levels of teacher-reported relational victimization). This calls into question what aspects of minority stressors actually lead to negative effects on mental health for LGB youth: the stigma and prejudice itself, or the subjective experiences of victimization and rejection by the LGB adolescent. Further research that dissects these mechanisms could shed more light on these processes. Finally, our study could serve to inform policy too. For instance, the fact that we detected mechanisms in line with the Minority Stress Framework (Meyer 2003) when our respondents were still in primary school, demonstrates the need for education of sexual diversity already in these stages of education, both of children and of parents.

## Conclusion

This study indicated that LGB adolescents are at an increased risk of depressive symptoms in comparison to their heterosexual counterparts. Disparities between LGB and heterosexual youth were found to be especially pronounced for girls and/or bisexuals. Our study adds to the literature by revealing that already at the age of 11, LB girls/bisexuals are at an increased risk of depressive symptoms compared to heterosexual youth. These differences were partly mediated by peer victimization and parental rejection. Such mechanisms have been demonstrated in adolescence (Pearson and Wilkinson 2013; Robinson et al. 2013); we extend existing research by demonstrating the presence of them as early as in late childhood. Another contribution is that we found that pubertal development was associated with an increase of depression disparities between LB and heterosexual youth. Even in a relatively LGB-friendly country as the Netherlands, LGB youth thus continue to find themselves in a setback position with regard to well-being. Further research and continued efforts to further increase the acceptance of diversity in sexual orientation are needed to change this.

## Electronic supplementary material

Supplementary material 1 (DOCX 30 kb)

## References

[CR1] Aalto-Setälä T, Marttunen M, Tuulio-Henriksson A, Poikolainen K, Lönnqvist J (2002). Depressive symptoms in adolescence as predictors of early adulthood depressive disorders and maladjustment. American Journal of Psychiatry.

[CR2] Achenbach T, Rescorla L (2001). ASEBA school-age forms and profiles.

[CR3] Achenbach T, Rescorla L (2003). Manual for the ASEBA adult forms & profiles.

[CR4] Acock A (2013). Discovering structural equation modeling using Stata.

[CR5] Allison PD (2003). Missing data techniques for structural equation modeling. Journal of Abnormal Psychology.

[CR6] Almeida J, Johnson R, Corliss H (2009). Emotional distress among LGBT youth: The influence of perceived discrimination based on sexual orientation. Journal of Youth and Adolescence.

[CR7] Baams L, Dubas JS, Overbeek G, van Aken MAG (2015). Transitions in body and behavior: A meta-analytic study on the relationship between pubertal development and adolescent sexual behavior. The Journal of Adolescent Health.

[CR8] Baams L, Grossman AH, Russell ST (2015). Minority stress and mechanisms of risk for depression and suicidal ideation among lesbian, gay, and bisexual youth. Developmental Psychology.

[CR9] Bentler P (1990). Comparative fit indexes in structural models. Psychological Bulletin.

[CR10] Birkett M, Newcomb ME, Mustanski B (2015). Does it get better? A longitudinal analysis of psychological distress and victimization in lesbian, gay, bisexual, transgender, and questioning youth. Journal of Adolescent Health.

[CR11] Bontempo DE, D’Augelli AR (2002). Effects of at-school victimization and sexual orientation on lesbian, gay, or bisexual youths’ health risk behavior. Journal of Adolescent Health.

[CR12] Bostwick WB, Boyd CJ, Hughes TL, McCabe SE (2010). Dimensions of sexual orientation and the prevalence of mood and anxiety disorders in the United States. American Journal of Public Health.

[CR13] Bostwick WB, Boyd CJ, Hughes TL, West BT, McCabe SE (2014). Discrimination and mental health among lesbian, gay, and bisexual adults in the United States. American Journal of Orthopsychiatry.

[CR14] Bouris A, Guilamo-Ramos V, Pickard A, Shiu C, Loosier PS, Dittus P (2010). A systematic review of parental influences on the health and well-being of lesbian, gay, and bisexual youth: Time for a new public health research and practice agenda. The Journal of Primary Prevention.

[CR15] Browne M, Cudeck R, Bollen KA, Long JS (1993). Alternative ways of assessing model fit. Testing structural equation models.

[CR16] Carr CL (2006). Bisexuality as a category in social research: Lessons from women’s gendered narratives. Journal of Bisexuality.

[CR17] Carr CL (2011). Women’s bisexuality as a category in social research, revisited. Journal of Bisexuality.

[CR18] Collier K, Bos H, Sandfort T (2013). Homophobic name-calling among secondary school students and its implications for mental health. Journal of Youth and Adolescence.

[CR19] Cox N, Berghe W, Dewaele A, Vincke J (2010). Acculturation strategies and mental health in gay, lesbian, and bisexual youth. Journal of Youth and Adolescence.

[CR20] Cyranowski JM, Frank E, Young E, Shear MK (2000). Adolescent onset of the gender difference in lifetime rates of major depression. Archives of General Psychiatry.

[CR21] D’Augelli AR, Pilkington NW, Hershberger SL (2002). Incidence and mental health impact of sexual orientation victimization of lesbian, gay, and bisexual youths in high school. School Psychology Quarterly.

[CR22] de Winter A, Oldehinkel A, Veenstra R, Brunnekreef JA, Verhulst FC, Ormel J (2005). Evaluation of non-response bias in mental health determinants and outcomes in a large sample of pre-adolescents. European Journal of Epidemiology.

[CR23] Deković M, ten Have M, Vollebergh WAM, Pels T, Oosterwegel A, Wissink IB (2006). The cross-cultural equivalence of parental rearing measure: EMBU-C. European Journal of Psychological Assessment.

[CR24] Diamond LM (2003). What does sexual orientation orient? A biobehavioral model distinguishing romantic love and sexual desire. Psychological Review.

[CR25] Dorn LD, Dahl RE, Woodward HR, Biro F (2006). Defining the boundaries of early adolescence: A user’s guide to assessing pubertal status and pubertal timing in research with adolescents. Applied Developmental Science.

[CR26] Enders C, Bandalos D (2001). The relative performance of full information maximum likelihood estimation for missing data in structural equation models. Structural Equation Modeling.

[CR27] Fish JN, Pasley K (2015). Sexual (minority) trajectories, mental health, and alcohol use: A longitudinal study of youth as they transition to adulthood. Journal of Youth and Adolescence.

[CR28] Friedman C, Leaper C (2010). Sexual-minority college women’s experiences with discrimination: Relations with identity and collective action. Psychology of Women Quarterly.

[CR29] Girgus JS, Yang K (2015). Gender and depression. Current Opinion in Psychology.

[CR30] Halpern CT, Udry JR, Campbell B, Suchindran C (1993). Testosterone and pubertal development as predictors of sexual activity: A panel analysis of adolescent males. Psychosomatic Medicine.

[CR31] Hatzenbuehler ML (2009). How does sexual minority stigma “get under the skin”? A psychological mediation framework. Psychological Bulletin.

[CR32] Herbenick D, Reece M (2010). Sexual behavior in the United States: Results from a national probability sample of men and women ages 14–94. The Journal of Sexual Medicine.

[CR33] Herdt G, McClintock M (2000). The magical age of 10. Archives of Sexual Behavior.

[CR34] Hill RM, Pettit JW, Lewinsohn PM, Seeley JR, Klein DN (2014). Escalation to major depressive disorder among adolescents with subthreshold depressive symptoms: Evidence of distinct subgroups at risk. Journal of Affective Disorders.

[CR35] Ho D, Imai K, King G, Stuart E (2007). Matching as nonparametric preprocessing for reducing model dependence in parametric causal inference. Political Analysis.

[CR36] Huisman M, Oldehinkel A, De Winter A, Minderaa RB, De Bildt A, Huizink AC (2008). Cohort profile: The Dutch “TRacking Adolescents” Individual lives’ Survey’; TRAILS. International Journal of Epidemiology.

[CR37] Institute of Medicine (2011). The health of lesbian, gay, bisexual, and transgender people.

[CR38] Janssens KAM, Rosmalen JGM, Ormel J, Verhulst FC, Hunfeld JAM, Mancl LA (2011). Pubertal status predicts back pain, overtiredness, and dizziness in American and Dutch adolescents. Pediatrics.

[CR39] Jiang Y, Perry D, Hesser J (2010). Adolescent suicide and health risk behaviors: Rhode Island’s 2007 youth risk behavior survey. American Journal of Preventive Medicine.

[CR40] Kite ME, Whitley BE (2003). Sex differences in attitudes toward homosexual persons, behaviors, and civil rights: A meta-analysis. Personality and Social Psychology Bulletin.

[CR41] Kuyper L, Bakker F, Vanwesenbeeck I (2006). Seksualiteit en seksuele gezondheid bij homo-en biseksuelen. Seksuele gezondheid in Nederland 2006.

[CR42] Kuyper L (2015). Jongeren en seksuele oriëntatie. Ervaringen van en opvattingen over homoseksuele, biseksuele en heteroseksuele jongeren.

[CR43] Kuyper L, Fokkema T (2011). Minority stress and mental health among Dutch LGBs: Examination of differences between sex and sexual orientation. Journal of Counseling Psychology.

[CR44] Larson R, Richards M (1989). Introduction: The changing life space of early adolescence. Journal of Youth and Adolescence.

[CR45] Lewis NM (2009). Mental health in sexual minorities: Recent indicators, trends, and their relationships to place in North America and Europe. Health & Place.

[CR46] Lubbers M, Jaspers E, Ultee W (2009). Primary and secondary socialization impacts on support for same-sex marriage after legalization in the Netherlands. Journal of Family Issues.

[CR47] Maguen S, Floyd FJ, Bakeman R, Armistead L (2002). Developmental milestones and disclosure of sexual orientation among gay, lesbian, and bisexual youths. Journal of Applied Developmental Psychology.

[CR48] Markus M, Lindhout I, Boer F, Hoogendijk T, Arrindell W (2003). Factors of perceived parental rearing styles: The EMBU-C examined in a sample of Dutch primary school children. Personality and Individual Differences.

[CR49] Marshal MP, Dermody SS, Cheong J, Burton CM, Friedman MS, Aranda F, Hughes TL (2013). Trajectories of depressive symptoms and suicidality among heterosexual and sexual minority youth. Journal of Youth and Adolescence.

[CR50] Marshal MP, Dietz L, Friedman M, Stall R, Smith H, McGinley J (2011). Suicidality and depression disparities between sexual minority and heterosexual youth: A meta-analytic review. Journal of Adolescent Health.

[CR51] McClintock M, Herdt G (1996). Rethinking puberty: The development of sexual attraction. Current Directions in Psychological Science.

[CR52] Meyer IH (2003). Prejudice, social stress, and mental health in lesbian, gay, and bisexual populations: Conceptual issues and research evidence. Psychological Bulletin.

[CR53] Meyer IH, Dietrich J, Schwartz S (2008). Lifetime prevalence of mental disorders and suicide attempts in diverse lesbian, gay, and bisexual populations. American Journal of Public Health.

[CR54] Morgan S, Harding D (2006). Matching estimators of causal effects prospects and pitfalls in theory and practice. Sociological Methods & Research.

[CR55] Mosher W, Chandra A, Jones J (2005). Sexual behavior and selected health measures: Men and women 15–44 years of age, United States, 2002.

[CR56] Mustanski B (2015). Future directions in research on sexual minority adolescent mental, behavioral, and sexual health. Journal of Clinical Child and Adolescent Psychology.

[CR57] Muthén BO, Curran PJ (1997). General longitudinal modeling of individual differences in experimental designs: A latent variable framework for analysis and power estimation. Psychological Methods.

[CR58] Needham BL (2012). Sexual attraction and trajectories of mental health and substance use during the transition from adolescence to adulthood. Journal of Youth and Adolescence.

[CR59] Needham BL, Austin EL (2010). Sexual orientation, parental support, and health during the transition to young adulthood. Journal of Youth and Adolescence.

[CR60] Newcomb ME, Mustanski B (2010). Internalized homophobia and internalizing mental health problems: A meta-analytic review. Clinical Psychology Review.

[CR61] Oldehinkel A, Rosmalen J, Buitelaar J, Hoek H, Ormel J, Raven D (2015). Cohort profile update: The TRacking Adolescents’ Individual Lives Survey (TRAILS). International Journal of Epidemiology.

[CR62] Oldehinkel A, Verhulst F, Ormel J (2011). Mental health problems during puberty: Tanner stage-related differences in specific symptoms. The TRAILS study. Journal of Adolescence.

[CR63] Pearson J, Wilkinson L (2013). Family relationships and adolescent well-being: Are families equally protective for same-sex attracted youth?. Journal of Youth and Adolescence.

[CR64] Petersen AC, Crockett L, Richards M, Boxer A (1988). A self-report measure of pubertal status: Reliability, validity, and initial norms. Journal of Youth and Adolescence.

[CR65] Petersen AC, Sarigiani PA, Kennedy RE (1991). Adolescent depression: Why more girls?. Journal of Youth and Adolescence.

[CR66] Pine D, Cohen E, Cohen P, Brook J (1999). Adolescent depressive symptoms as predictors of adult depression: Moodiness or mood disorder?. American Journal of Psychiatry.

[CR67] Pramaggiore M, Storr M (2002). Extracts from epistemologies of the fence. Bisexuality: A critical reader.

[CR68] Preacher KJ, Hayes AF (2008). Asymptotic and resampling strategies for assessing and comparing indirect effects in multiple mediator models. Behavior Research Methods.

[CR69] Puckett JA, Woodward EN, Mereish EH, Pantalone DW (2015). Parental rejection following sexual orientation disclosure: Impact on internalized homophobia, social support, and mental health. LGBT Health.

[CR70] Robinson JP, Espelage DL, Rivers I (2013). Developmental trends in peer victimization and emotional distress in LGB and heterosexual youth. Pediatrics.

[CR71] Rothman EF, Sullivan M, Keyes S, Boehmer U (2012). Parents’ supportive reactions to sexual orientation disclosure associated with better health: Results from a population-based survey of LGB adults in Massachusetts. Journal of Homosexuality.

[CR72] Russell ST, Toomey RB, Ryan C, Diaz RM (2014). Being out at school: The implications for school victimization and young adult adjustment. American Journal of Orthopsychiatry.

[CR73] Rust PC (2000). Bisexuality in the United States: A social science reader.

[CR74] Rust PC (2002). Bisexuality: The state of the union. Annual Review of Sex Research.

[CR75] Ryan C, Huebner D, Diaz RM, Sanchez J (2009). Family rejection as a predictor of negative health outcomes in white and Latino lesbian, gay, and bisexual young adults. Pediatrics.

[CR76] Saewyc EM (2011). Research on adolescent sexual orientation: Development, health disparities, stigma, and resilience. Journal of Research on Adolescence.

[CR77] Saluja G, Iachan R, Scheidt PC, Overpeck MD, Sun W, Giedd JN (2004). Prevalence of and risk factors for depressive symptoms among young adolescents. Archives of Pediatrics and Adolescent Medicine.

[CR78] Savin-Williams RC (2006). Who’s Gay? Does It Matter?. Current Directions in Psychological Science.

[CR79] Savin-Williams RC, Diamond LM (2000). Sexual identity trajectories among sexual-minority youths: Gender comparisons. Archives of Sexual Behavior.

[CR80] Shirtcliff E, Dahl R, Pollak S (2009). Pubertal development: Correspondence between hormonal and physical development. Child Development.

[CR81] Smith EA, Udry JR, Morris NM (1985). Pubertal development and friends: A biosocial explanation of adolescent sexual behavior. Journal of Health and Social Behavior.

[CR82] StataCorp LP (2013). Stata: Release 13.

[CR83] Stuart EA (2010). Matching methods for causal inference: A review and a look forward. Statistical Science.

[CR84] Takács J, Szalma I (2013). How to measure homophobia in an international comparison. Družboslovne Razprave.

[CR85] Teasdale B, Bradley-Engen MS (2010). Adolescent same-sex attraction and mental health: The role of stress and support. Journal of Homosexuality.

[CR86] Ueno K, Vaghela P, Ritter LJ (2014). Sexual orientation, internal migration, and mental health during the transition to adulthood. Journal of Health and Social Behavior.

[CR87] Van Bergen DD, Bos HMW, Van Lisdonk J, Keuzenkamp S, Sandfort TGM (2013). Victimization and suicidality among Dutch lesbian, gay, and bisexual youths. American Journal of Public Health.

[CR88] Van Beusekom, G., Baams, L., Bos, H. M. W., Overbeek, G., & Sandfort, T. G. M. (2016). Gender nonconformity, homophobic peer victimization, and mental health: How same-sex attraction and biological sex matter. *Journal of Sex Research*, *53*(1), 1–11. 10.1080/00224499.2014.993462 PMC5872829 26099017

[CR89] Van den Akker H, Van der Ploeg R, Scheepers P (2013). Disapproval of homosexuality: Comparative research on individual and national determinants of disapproval of homosexuality in 20 European countries. Journal of Public Opinion Research.

[CR90] van Lang NDJ, Ferdinand RF, Oldehinkel AJ, Ormel J, Verhulst FC (2005). Concurrent validity of the DSM-IV scales affective problems and anxiety problems of the youth self-report. Behaviour Research and Therapy.

[CR91] Wang J, Dey M, Soldati L, Weiss M, Gmel G, Mohler-Kuo M (2014). Psychiatric disorders, suicidality, and personality among young men by sexual orientation. European Psychiatry.

[CR92] Weinberg M, Williams C, Pryor D (1994). Dual attraction: Understanding bisexuality.

[CR93] Williams T, Connolly J, Pepler D, Craig W (2005). Peer victimization, social support, and psychosocial adjustment of sexual minority adolescents. Journal of Youth and Adolescence.

[CR94] Wu W, West SG, Hughes JN (2008). Effect of retention in first grade on children’s achievement trajectories over 4 years: A piecewise growth analysis using propensity score matching. Journal of Educational Psychology.

[CR95] Wu W, West SG, Hughes JN (2010). Effect of grade retention in first grade on psychosocial outcomes. Journal of Educational Psychology.

