# Supplementary material for: Disparities in Depressive Symptoms Between Heterosexual and Lesbian, Gay, and Bisexual Youth in a Dutch Cohort: The TRAILS Study
Source: J Youth Adolesc. 2016 Jan 9;45:440–56. doi: 10.1007/s10964-015-0403-0 (PMC4749655; doi:10.1007/s10964-015-0403-0)
Supplement: Supplementary file 1 — Supplementary material 1 (DOCX 30 kb) [file 10964_2015_403_MOESM1_ESM.docx]

**Appendix A: Propensity Score Matching Procedure**

We employed propensity score matching in order to balance the distribution of background factors that enhance the probability of depressive symptoms, yet are unrelated to one’s sexual orientation and the stigma and prejudice related to it, which we envision to lead to disparities in depressive symptoms between heterosexual and LGB youth.

Propensity score estimates were used estimating the probability of being LGB (or bisexual) for all 1738 respondents for whom information on sexual orientation was available. Multiple neighbors within caliper matching with resampling of matched control (heterosexual) cases were used. We allowed for up to 10 potential neighbors for every LGB respondents. When choosing a caliper, we sought for a caliper size that made us achieve balance without losing substantial numbers of “treatment” (LGB) respondents due to absence of heterosexual respondents that were similar enough to them (Morgan and Harding 2006). A caliper of 0.025 points difference on the propensity score fulfilled this aim. Our analyses were consequently performed on the matched and weighted groups (Wu et al. 2008, 2010).

Because we performed separate analyses for boys, girls and heterosexuals versus bisexuals, we also performed separate propensity score matching procedures for all of these subgroups. We chose to match on variables based on background characteristics of respondents measured at the first wave, or retrospective accounts of characteristics of the respondent’s life that predated wave one (mostly based on parental reports). The following matching variables were used (see also Heininga et al. 2015):

**Childhood events.** A variable was created that summed detrimental childhood events, by summing the following events, measured at wave 1: parental divorce, death of household member, severe illness of sibling, severe (physical or mental) illness of father and/or mother. Childhood events were measured through parental reports.

**Long-term difficulties.** A variable was created measuring the presence of long-term difficulties (mostly within the family-context), by summing the following items: Chronicle disease of respondent or household member, problems with house (e.g. too noisy; to small), problems in neighborhood (e.g. vandalism, unsafety), financial difficulties within the family, protracted conflicts between family members. Long-term difficulties were measured through parental reports.

**Perinatal complications.** An index of perinatal complications was composed by adding the scores for the difficulty if the pregnancy and the delivery (each rated on a four-point scales ranging from 0 = not at all to 3 = very difficult) plus one point added for each of the following possibilities: incubation following birth, oxygen administration after birth, jaundice, blood transfusion after birth, maternal (physical, social, or psychological) problems in the first month after birth. Perinatal complications were measured through parental reports.

**Parental SES.** A composite measure of parental socio-economic status was created by adding the Z-scores of parental occupational status (ISCO-88), parental education and parental income (Amone-P’Olak et al. 2009).

**Intelligence.** At wave one, an index of respondents’ intelligence was created by combining two subsets of the WISC-R (Silverstein 1975). Scores on subsets were standardized and age differences at wave one were taken into account.

**Early childhood (age 0-5) stressfulness of life.** At wave 2, parents were asked to rate the stressfulness of the life of their parents between ages 0 and 5 on an 11-point scale, ranging from 0=not at all to 10=very much.

**Family depression levels.** A continuous scale was created that measured the depression and anxiety disorders of one or both parents and was measured at the wave one parent interview (Veenstra et al. 2005).

**Missing data dummy.** In order to prevent cases from dropping out due to missing information on the propensity score variables, we used mean imputation for matching variables that had non-negligible proportions of missing data (childhood events, long-term difficulties, parental SES, early life stress). Furthermore, a dummy was created that indicated whether or not information on matching variables was originally missing. This dummy variable was also used as a matching variable. Thereby, potentially informative differences in missingness on matching variables between heterosexual and LGB respondents were taken into account when matching respondents.

**Sex.** Respondents’ sex was used as matching variable when matching bisexual respondents with comparable heterosexual respondents.

**Results**

Tables A1 to A3 show that differences in standardized propensity scores between LGB and heterosexual respondents were moderate, yet highly statistically significant before matching (boys: -0.36, *p* < .001; girls: -0.65, *p* < .001; bisexuals versus heterosexuals: -0.66, *p* < .001). After matching, differences in standardized propensity scores were very close to zero and non-significant (boys: -0.02, *n.s.*; girls: -0.01, *n.s.*; bisexuals versus heterosexuals: -0.01, *n.s.*). This suggests that balance between our LGB respondents and the matched heterosexual respondents was achieved and that differences with regard to depressive symptoms and explanatory mechanisms for these disparities, cannot be attributed to differences in the matching variables (Stuart 2010). As for the differences on the individual matching variables, it holds that differences were mostly smaller after matching than prior to matching. Furthermore, we see that for girls, there were significant differences in intelligence before matching. For bisexuals compared to heterosexuals, there were both significant differences in intelligence and the proportion of females before matching. All these differences were no longer significant after matching. Both in terms of parameter estimate differences, as well in terms of significance, propensity score matching thus substantially improved balance.

**Table A1. Balance boys**

|  | Before matching | | | After matching | | |
| --- | --- | --- | --- | --- | --- | --- |
|  | Heterosexual (n=727) | LGB (n=58) | difference | Heterosexual (n=380) | LGB (n=57) | difference |
| Standardized propensity score | -0.027 | 0.330 | -0.356** | 0.311 | 0.330 | -0.019 |
| Childhood events | -0.034 | -0.001 | -0.033 | 0.0102 | -0.007 | 0.018 |
| Longterm difficulties | -0.014 | 0.037 | -0.051 | 0.015 | 0.047 | -0.032 |
| Perinatal problems | 10.048 | 10.224 | -0.176 | 10.285 | 10.245 | 0.040 |
| Parental SES (centered) | 0.018 | -0.081 | 0.099 | -0.032 | -0.094 | 0.062 |
| Intelligence | 100.62 | 100.02 | 0.60 | 101.264 | 100.43 | 0.826 |
| Early life stress | 0.098 | -0.032 | 0.131 | -0.199 | -0.149 | -0.051 |
| Parental depressive symptoms | 0.526 | 0.402 | 0.122 | 0.398 | 0.403 | -0.004 |
| Missing data matching variables | 0.109 | 0.086 | 0.022 | 0.096 | 0.088 | 0.009 |

**Table A2. Balance girls**

|  | Before matching | | | After matching | | |
| --- | --- | --- | --- | --- | --- | --- |
|  | Heterosexual (n=860) | LGB (n=93) | difference | Heterosexual (n=486) | LGB (n=90) | difference |
| Standardized propensity score | -0.064 | 0.587 | -0.651*** | 0.518 | 0.524 | -0.006 |
| Childhood events | 0.034 | -0.046 | 0.081 | -0.063 | -0.047 | -0.015 |
| Longterm difficulties | -0.005 | 0.129 | -0.133 | 0.107 | 0.144 | -0.037 |
| Perinatal problems | 0.991 | 0.914 | 0.077 | 0.795 | 0.911 | -0.116 |
| Parental SES (centered) | -0.001 | -0.087 | 0.087 | -0.099 | -0.072 | -0.026 |
| Intelligence | 96.99 | 103.63 | -6.66*** | 102.95 | 103.31 | -0.36 |
| Early life stress | -0.076 | -0.048 | -0.027 | -0.096 | -0.030 | -0.066 |
| Parental depressive symptoms | 0.548 | 0.575 | -0.027 | 0.530 | 0.566 | -0.035 |
| Missing data matching variables | 0.119 | 0.086 | 0.033 | 0.078 | 0.078 | 0.000 |

**Table A3. Balance heterosexuals versus bisexuals**

|  | Before matching | | | After matching | | |
| --- | --- | --- | --- | --- | --- | --- |
|  | Heterosexual (n=1587) | LGB (n=117) | difference | Heterosexual (n=744) | LGB (n=112) | difference |
| Standardized propensity score | -0.045 | 0.617 | -0.663*** | 0.508 | 0.516 | -0.008 |
| Childhood events | 0.003 | -0.025 | 0.028 | -0.031 | -0.022 | -0.009 |
| Longterm difficulties | -0.009 | 0.105 | -0.114 | 0.135 | 0.075 | 0.060 |
| Perinatal problems | 10.017 | 10.025 | -0.008 | 10.053 | 10.018 | 0.036 |
| Parental SES (centered) | 0.008 | -0.100 | 0.108 | -0.080 | -0.098 | 0.018 |
| Intelligence | 98.65 | 102.42 | -3.77** | 102.57 | 102.24 | 0.33 |
| Early life stress | 0.004 | -0.042 | 0.046 | -0.096 | -0.093 | -0.003 |
| Parental depressive symptoms | 0.538 | 0.540 | -0.002 | 0.580 | 0.555 | 0.025 |
| Missing data matching variables | 0.114 | 0.111 | 0.003 | 0.107 | 0.107 | -0.000 |
| Gender(1=female) | 0.542 | 0.701 | -0.159*** | 0.676 | 0.696 | -0.021 |

**References**

Amone-P’Olak, K., Ormel, J., Huisman, M., Verhulst, F. C., Oldehinkel, A. J., & Burger, H. (2009). Life stressors as mediators of the relation between socioeconomic position and mental health problems in early adolescence: the TRAILS study. *Journal of the American Academy of Child & Adolescent Psychiatry*, *48*(10), 1031–1038.

Heininga, V., Oldehinkel, A., Veenstra, R., & Nederhof, E. (2015). I just ran a thousand analyses: benefits of multiple testing in understanding equivocal evidence on gene-environment interactions. *PloS One*, *10*(5), e0125383.

Morgan, S., & Harding, D. (2006). Matching estimators of causal effects prospects and pitfalls in theory and practice. *Sociological Methods & Research*, *35*(1), 3–60.

Silverstein, A. (1975). Validity of WISC-R short forms. *Journal of Clinical Psychology*, *31*, 696–697.

Stuart, E. A. (2010). Matching methods for causal inference: a review and a look forward. *Statistical Science*, *25*(1), 1–21.

Veenstra, R., Lindenberg, S., Verhulst, F. C., & Ormel, J. (2005). Bullying and victimization in elementary schools: a comparison of bullies, victims, bully/victims, and uninvolved preadolescents. *Developmental Psychology*, *41*(4), 672–682.

Wu, W., West, S. G., & Hughes, J. N. (2008). Effect of retention in first grade on children’s achievement trajectories over 4 years: a piecewise growth analysis using propensity score matching. *Journal of Educational Psychology*, *100*(4), 727–740.

Wu, W., West, S. G., & Hughes, J. N. (2010). Effect of grade retention in first grade on psychosocial outcomes. *Journal of Educational Psychology*, *102*(1), 135–152.
